# A laser pulse impactful on a half-space using the modified TPL G–N models

**DOI:** 10.1038/s41598-020-61249-y

**Published:** 2020-03-10

**Authors:** Ashraf M. Zenkour, Daoud S. Mashat

**Affiliations:** 1 Department of Mathematics, Faculty of Science, King Abdulaziz University, P.O. Box 80203, Jeddah, 21589 Saudi Arabia; 2 Department of Mathematics, Faculty of Science, Kafrelsheikh University, Kafrelsheikh, 33516 Egypt

**Keywords:** Energy science and technology, Engineering, Materials science, Mathematics and computing, Nanoscience and technology

## Abstract

This article aims to investigate the wave propagation of generalized thermoelastic half-plane under the effect of thermal loading due to laser pulse with and without energy dissipation. The normal mode method is proposed to solve the problem and get numerical results for the field quantities. The outcomes of the physical quantities have been illustrated graphically and reported to compare the simple Green–Naghdi II and III and their modified single-, dual-, and three-phase-lag models. The graphical outcomes indicate that the different types of Green–Naghdi models with thermal relaxations have great effects on the temperature, displacements, dilatation and stresses.

## Introduction

Coupling between mechanical and thermal fields have not occurred in the classical theory of thermoelasticity and so one needs more coupled and generalized theories. The coupled thermoelasticity theory of Biot^1^ thinks about the trading of mechanical energy and the thermal energy but one still needs the generalized theories. One of the most important generalized thermoelasticity theories is the theory of Green–Naghdi (G–N)^2–4^. This theory is a consistent one that considers elastic and thermal waves associated with the second sound. A lot of researchers dealt with various theoretical and practical features in thermoelasticity, in the context of the G–N models of type II or/and of type III.

Excitation of thermoelastic waves by a pulsed laser in a continuum is of incredible enthusiasm because of broad utilization of pulsed laser advancements in material handling and non-destructive recognizing and characterization. At the point when the continuum is illuminated with a laser pulse, assimilation of the laser pulse brings about a restricted temperature increment, which thus causes thermal extension and creates a thermoelastic wave in the medium. Deswal *et al*.^5^ studied the vibrations induced by a laser beam in the context of generalized magneto-thermoelasticity for isotropic and homogeneous elastic solids under G–N model in the *x*-*z* plane. Youssef and El-Bary^6^ derived the induced temperature and stress fields in an elastic half-space heated by a non-Gaussian laser beam with the pulse in the context of different coupled thermoelasticity theories. Othman *et al*.^7^ studied the rotation of initially stressed thermoelastic half-space with voids under thermal loading due to laser pulse in the context of G–N theory. Zenkour and Abouelregal^8^ investigated the vibration analysis of a nanobeam under a sinusoidal pulse varying heat in the context of a unified generalized nonlocal thermoelasticity theory with dual-phase-lag (DPL).

Othman and Tantawi^9^ investigated the impact of the gravitational field on a 2D thermoelastic solid affected by thermal loading because of a laser pulse. Abbas and Marin^10^ considered the problem of a 2D thermoelastic half-space by pulsed laser heating with regards to the generalized thermoelastic theory with one relaxation time. Ailawalia *et al*.^11^ presented the 2D deformation under the impact of laser pulse heating in a thermo microstretch elastic medium at the interface of thermoelastic solid in the context of G–N theory. Othman and Marin^12^ discussed the wave propagation of generalized thermoelastic half-space with voids under the impact of thermal loading because of a laser pulse with energy dissipation. Mondal *et al*.^13^ analyzed the effect of the laser pulse as a heat source utilizing a memory-dependent derivative with regards to three thermoelastic theories. Ailawalia and Singla^14^ dealt with the 2D deformation of laser pulse heating in a thermoelastic micro-elongated layer immersed in an infinite non-viscous fluid. Othman and Abd-Elaziz^15^ studied the impact of thermal loading because of a laser pulse in generalized thermoelastic half-space with voids in a DPL theory.

This article presents the temperature, displacements and stresses of a thermoelastic half-space under the impact of thermal loading because of a laser pulse. The material of the present thermoelastic half-space is homogeneous and isotropic and the medium itself is heated by a non-Gaussian laser beam with pulse duration. The normal mode method is proposed to obtain the numerical outcomes for the temperature, displacements, dilatation and stresses. These variables have been illustrated graphically by comparison between Green–Naghdi theory of both types II and III to show the advantages presented by the present modified models.

## Different thermoelasticity models

In what follows we present a unified three-phase lag (TPL) Green–Naghdi heat conduction equation. Let the temperature change is small enough compared to the reference temperature, that is *θ* → *T*_0_. So, the heat conduction equation can be simplified as (Zenkour^16–21^)

1 $$k{ {\mathcal L} }_{1}({\nabla }^{2}\theta )={ {\mathcal L} }_{2}(\rho {C}_{e}\theta +\gamma {T}_{0}e)-{ {\mathcal L} }_{0}(\rho {Q}_{0}).$$

In addition, the time differential operators ${ {\mathcal L} }_{i}$ (*i* = 0, 1, 2) are given by

2 $$\begin{array}{ccc}{{\mathcal{L}}}_{1} & = & \left(1+{\sum }_{n=1}^{N}\frac{{\tau }_{\theta }^{n}}{n!}\frac{{{\rm{\partial }}}^{n}}{{\rm{\partial }}{t}^{n}}\right)\frac{{\rm{\partial }}}{{\rm{\partial }}t}+\frac{\epsilon {k}^{\ast }}{k}\left(1+{\sum }_{n=1}^{N}\frac{{\tau }_{\vartheta }^{n}}{n!}\frac{{{\rm{\partial }}}^{n}}{{\rm{\partial }}{t}^{n}}\right),\\ {{\mathcal{L}}}_{0} & = & \left(1+{\sum }_{n=1}^{N}\frac{{\tau }_{q}^{n}}{n!}\frac{{{\rm{\partial }}}^{n}}{{\rm{\partial }}{t}^{n}}\right)\frac{{\rm{\partial }}}{{\rm{\partial }}t},\,{{\mathcal{L}}}_{2}={{\mathcal{L}}}_{0}\frac{{\rm{\partial }}}{{\rm{\partial }}t},\end{array}$$

in which *ϵ* is a dimensionless key number, may equal only to zero or one. Also, the thermal relaxation time parameters *τ*_*q*_, *τ*_*θ*_ and *τ*_*ϑ*_ are the thermal memories with $0\le {\tau }_{\vartheta } < {\tau }_{\theta } < {\tau }_{q}$. Equation (1) is more general when *N* has different integers greater than zero. Some special cases may be obtained from the above relations as

(i) TPL G–N III model (*ϵ* = 1, *N* ≥ 1).

(ii) DPL G–N III model (*ϵ* = 1, *τ*_*θ*_ = 0, *N* ≥ 1).

(iii) SPL G–N III model (*ϵ* = 1, *τ*_*θ*_ = *τ*_*ϑ*_ = 0, *N* ≥ 1).

(iv) DPL G–N II model (*ϵ* = 0, *N* ≥ 1):

3 $$k\left(1+{\sum }_{n=1}^{N}\frac{{\tau }_{\theta }^{n}}{n!}\frac{{{\rm{\partial }}}^{n}}{{\rm{\partial }}{t}^{n}}\right)({{\rm{\nabla }}}^{2}\theta )=\left(1+{\sum }_{m=1}^{N}\frac{{\tau }_{q}^{m}}{m!}\frac{{{\rm{\partial }}}^{m}}{{\rm{\partial }}{t}^{m}}\right)\left[\frac{{\rm{\partial }}}{{\rm{\partial }}t}(\rho {C}_{e}\theta +\gamma {T}_{0}e)-\rho {Q}_{0}\right].$$

(v) SPL G–N II model (∈ = 0, *τ*_*θ*_ = 0, *N* ≥ 1):

4 $$k({{\rm{\nabla }}}^{2}\theta )=\left(1+{\sum }_{m=1}^{N}\frac{{\tau }_{q}^{m}}{m!}\frac{{{\rm{\partial }}}^{m}}{{\rm{\partial }}{t}^{m}}\right)\left[\frac{{\rm{\partial }}}{{\rm{\partial }}t}(\rho {C}_{e}\theta +\gamma {T}_{0}e)-\rho {Q}_{0}\right].$$

(vi) Simple G–N III model (∈ = 1, *τ*_*q*_ = *τ*_*θ*_ = *τ*_*ϑ*_ = 0):

5 $$\left(k\frac{{\rm{\partial }}}{{\rm{\partial }}t}+{k}^{\ast }\right){{\rm{\nabla }}}^{2}\theta =\frac{{\rm{\partial }}}{{\rm{\partial }}t}\left[\frac{{\rm{\partial }}}{{\rm{\partial }}t}(\rho {C}_{e}\theta +\gamma {T}_{0}e)-\rho {Q}_{0}\right].$$

(vii) Simple G–N II model (*k* → 0, *ϵ* = 1, *τ*_*q* _= 0):

6 $${k}^{\ast }{{\rm{\nabla }}}^{2}\theta =\frac{{\rm{\partial }}}{{\rm{\partial }}t}\left[\frac{{\rm{\partial }}}{{\rm{\partial }}t}(\rho {C}_{\vartheta }\theta +\gamma {T}_{0}e)-\rho {Q}_{0}\right],$$

or

(viii) Simple G–N II model (*ϵ* = 0, *τ*_*q*_ = *τ*_*θ*_ = 0):

7 $$k{\nabla }^{2}\theta =\frac{\partial }{\partial t}(\rho {C}_{\vartheta }\theta +\gamma {T}_{0}e)-\rho {Q}_{0}.$$

It is to be noted that Eqs. (6) and (7) represent two forms of the simple G–N II model, the first is in terms of the rate of thermal conductivity k^*^ while the second is in terms of the heat conductivity coefficient *k*. A lot of investigators have dealt with the simple G–N II and III models while other investigators have dealt with the TPL G–N III model (*N* = 1)^22–41^. All these models are presented without the higher-order time derivatives as those presented in this study.

## Basic equations

**Figure 1:**
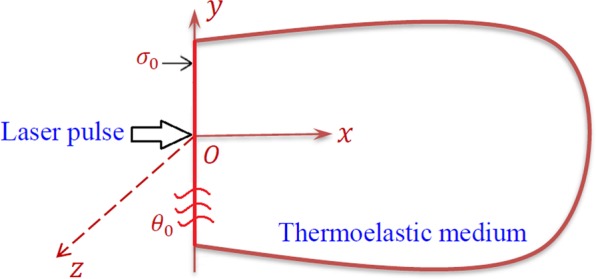
Geometry of the thermoelastic half-space.

Consider a thermoelastic problem of a half-space medium as shown in Fig. 1 in with regards to the multi-dual-phase-lag theory. The present half-space is characterized in the region Ψ as follows:

8 $$\Psi =\{(x,\,y,\,z)\,:0\le x < \infty ,\,-\infty  < y < \infty ,\,z=0\}.$$

Here, all variables will be depending on *t*, *x* and *y*. So, our analysis has been taken in the 2D *xy*-plane. The displacement vector can be taken in the form $\overrightarrow{u}=(u,v,0)$, where *u* and *v* are the horizontal and vertical components. Thus, the displacements *u*_*i*_ will be

9 $${u}_{1}=u(x,y,t),\,{u}_{2}=v(x,y,t),\,{u}_{3}=0.$$

The equations of motion are expressed as

10 $${\sigma }_{ij,j}+{F}_{i}=\rho {\ddot{u}}_{i}.$$

The constitutive equations will be simplified to

11 $${\sigma }_{ij}=2\mu {e}_{ij}+(\lambda e-\gamma \theta ){\delta }_{ij}.$$

For the present problem one can summarized the governing equations in the form

12 $$\begin{array}{rcl}\mu {\nabla }^{2}u+(\lambda +\mu )\frac{\partial e}{\partial x}-\gamma \frac{\partial \theta }{\partial x} & = & \rho \frac{{\partial }^{2}u}{\partial {t}^{2}},\\ \mu {\nabla }^{2}v+(\lambda +\mu )\frac{\partial e}{\partial y}-\gamma \frac{\partial \theta }{\partial y} & = & \rho \frac{{\partial }^{2}v}{\partial {t}^{2}},\end{array}$$

13 $$k{ {\mathcal L} }_{1}({\nabla }^{2}\theta )={ {\mathcal L} }_{2}(\rho {C}_{e}\theta +\gamma {T}_{0}e)-{ {\mathcal L} }_{0}(\rho {Q}_{0}).$$

The plate surface is lit up by the laser pulse given by the heat input

14 $${Q}_{0}=\frac{{I}_{0}{\gamma }_{0}}{2\pi {r}^{2}}\exp \left(-\frac{{y}^{2}}{{r}^{2}}-{\gamma }_{0}x\right)\,{f}_{0}(t).$$

The temporal profile *f*(*t*) can be defined as

15 $${f}_{0}(t)=\frac{t}{{t}_{0}^{2}}\exp \left(-\frac{t}{{t}_{0}}\right).$$

In the accompanying relations, it is advantageous to report the dimensionless variables in the form:

16 $$\begin{array}{c}\{x{\rm{{\prime} }},y{\rm{{\prime} }},r{\rm{{\prime} }}\}=\frac{\{x,y,r\}}{\eta },\,\,\{u{\rm{{\prime} }},v{\rm{{\prime} }}\}=\frac{\lambda +2\mu }{\eta \gamma {T}_{0}}\{u,v\},\,\,\,e{\rm{{\prime} }}=\frac{\lambda +2\mu }{\gamma {T}_{0}}e,\\ \{t{\rm{{\prime} }},{\tau }_{\vartheta }{\rm{{\prime} }},{\tau }_{\theta }{\rm{{\prime} }},{\tau }_{q}{\rm{{\prime} }}\}=\frac{{c}_{0}}{\eta }\{t,{\tau }_{\vartheta },{\tau }_{\theta },{\tau }_{q}\},\,\,\,{\gamma }_{0}{\rm{{\prime} }}=\eta {\gamma }_{0},\,\,\,{\sigma }_{ij}{\rm{{\prime} }}=\frac{{\sigma }_{ij}}{\gamma {T}_{0}},\,\,\,\theta {\rm{{\prime} }}=\frac{\theta }{{T}_{0}}\end{array}$$

where $({k}^{\ast }){\rm{{\prime} }}=\frac{\eta {k}^{\ast }}{{c}_{0}k}$, ${Q}_{0}{\rm{{\prime} }}=\frac{{c}_{0}\rho {Q}_{0}}{k{T}_{0}}$, ${c}_{0}^{2}=\frac{\lambda +2\mu }{\rho }$ and $\eta =\frac{k}{\rho {c}_{0}{C}_{e}}$. All governing equations, with the above non-dimensions, are reduced to (dropping the dashed for comfort)

17 $$\begin{array}{ccc}{\sigma }_{11} & = & \frac{{\rm{\partial }}u}{{\rm{\partial }}x}+{c}_{1}\frac{{\rm{\partial }}v}{{\rm{\partial }}y}-\theta ,\\ {\sigma }_{12} & = & {c}_{1}\left(\frac{{\rm{\partial }}v}{{\rm{\partial }}x}+\frac{{\rm{\partial }}u}{{\rm{\partial }}y}\right),\\ {\sigma }_{22} & = & {c}_{1}\frac{{\rm{\partial }}u}{{\rm{\partial }}x}+\frac{{\rm{\partial }}v}{{\rm{\partial }}y}-\theta ,\end{array}$$

18 $$\begin{array}{rcl}{\nabla }^{2}u+{c}_{2}\frac{\partial e}{\partial x}-{c}_{3}\frac{\partial \theta }{\partial x} & = & {c}_{3}\frac{{\partial }^{2}u}{\partial {t}^{2}},\\ {\nabla }^{2}v+{c}_{2}\frac{\partial e}{\partial y}-{c}_{3}\frac{\partial \theta }{\partial y} & = & {c}_{3}\frac{{\partial }^{2}v}{\partial {t}^{2}},\end{array}$$

19 $${ {\mathcal L} }_{1}({\nabla }^{2}\theta )={ {\mathcal L} }_{2}(\theta +{c}_{4}e)+{\bar{Q}}_{0},$$

where

20 $$\begin{array}{c}{c}_{1}=\frac{\lambda }{\lambda +2\mu },\,\,{c}_{2}=\frac{\lambda +\mu }{\mu },\,\,{c}_{3}=\frac{\lambda +2\mu }{\mu },\,\,{c}_{4}=\frac{{T}_{0}{\gamma }^{2}}{{c}_{0}^{2}{C}_{e}{\rho }^{2}},\\ {\bar{Q}}_{0}=\frac{{I}_{0}{\gamma }_{0}{q}_{t}}{2\pi {r}^{2}{t}_{0}^{3}}\,\exp \left(-\frac{{y}^{2}}{{r}^{2}}-\frac{t}{{t}_{0}}\right)\exp (\,-\,{\gamma }_{0}x),\\ {q}_{t}={\sum }_{n=0}^{N}\frac{{\tau }_{q}^{n}[t-(n+1){t}_{0}]}{{(-{t}_{0})}^{n}\,n!}.\end{array}$$

## Solution of the problem

To obtain the total solutions of the physical amounts, the normal mode method is applied. The physical fields in this system are shown as

21 $$\{u,v,\theta ,{\sigma }_{ij}\}(x,y,t)=\{{u}^{\ast },{v}^{\ast },{\theta }^{\ast },{\sigma }_{ij}^{\ast }\}(x)\,\exp (\omega t+{\rm{i}}by),$$

where *ω* = *ω*_0_ + i*ω*_1_ in which *ω*_0_ and *ω*_1_ are constants, ${\rm{i}}=\sqrt{-1}$, *b* represents the wave number in *y*-direction while the values of u^*^(*x*), v^*^(*x*), θ^*^(*x*) and ${\sigma }_{ij}^{\ast }(x)$ are the amplitudes of the quantities *u*(*x*), *v*(*x*), *θ*(*x*) and *σ*_*ij*_(*x*), respectively.

Applying the normal mode method on Eqs. (18) and (19), we have the following system of three ordinary homogeneous differential equations:

22 $$({{\mathscr{D}}}^{2}-{c}_{5}){u}^{\ast }+{c}_{6}{v}^{\ast }-{c}_{7}{\mathscr{D}}{\theta }^{\ast }=0,$$

23 $$({{\mathscr{D}}}^{2}-{c}_{8}){v}^{\ast }+{c}_{9}{\mathscr{D}}{u}^{\ast }-{c}_{10}{\theta }^{\ast }=0,$$

24 $$({{\mathscr{D}}}^{2}-{c}_{11}){\theta }^{\ast }-{c}_{12}{\mathscr{D}}{u}^{\ast }-{c}_{13}{v}^{\ast }=g(y,t)\,\exp (\,-\,{\gamma }_{0}x),$$

where

25 $$\begin{array}{c}{c}_{5}=\frac{{b}^{2}+{c}_{3}{\omega }^{2}}{1+{c}_{2}},\,{c}_{6}=\frac{{\rm{i}}b{c}_{2}}{1+{c}_{2}},\,{c}_{7}=\frac{{c}_{3}}{1+{c}_{2}},\,{c}_{8}={b}^{2}(1+{c}_{2})+{c}_{3}{\omega }^{2},\\ \{{c}_{9},{c}_{10}\}={\rm{i}}b\{{c}_{2},{c}_{3}\},\,{c}_{11}={b}^{2}+\frac{{\varpi }_{2}}{{\varpi }_{1}},\,{c}_{12}={c}_{4}\frac{{\varpi }_{2}}{{\varpi }_{1}},\,{c}_{13}={\rm{i}}b{c}_{12},\,{\mathscr{D}}=\frac{{\rm{d}}}{{\rm{d}}x},\\ g(y,t)=\frac{{I}_{0}{\gamma }_{0}{q}_{t}}{2\pi {r}^{2}{t}_{0}^{3}{\varpi }_{1}}\exp \left(-\frac{{y}^{2}}{{r}^{2}}-{\rm{i}}by-\frac{1+\omega {t}_{0}}{{t}_{0}}t\right),\end{array}$$

in which

26 $${\varpi }_{1}=\omega \left(1+\mathop{\sum }\limits_{n=1}^{N}\frac{{\tau }_{\theta }^{n}}{n!}{\omega }^{n}\right)+\frac{\epsilon {k}^{\ast }}{k}\left(1+\mathop{\sum }\limits_{n=1}^{N}\frac{{\tau }_{\vartheta }^{n}}{n!}{\omega }^{n}\right),\,{\varpi }_{2}={\omega }^{2}\left(1+\mathop{\sum }\limits_{n=1}^{N}\frac{{\tau }_{q}^{n}}{n!}{\omega }^{n}\right).$$

The system of differential Eqs. (22)–(24) may be given in a unified form as

27 $$({D}^{6}-{B}_{2}{D}^{4}+{B}_{1}{D}^{2}-{B}_{0})\{{u}^{\ast },\,{v}^{\ast },{\theta }^{\ast }\}(x)=\{{Q}_{1},{Q}_{2},{Q}_{3}\}\,g(y,t)\exp (-{\gamma }_{0}x),$$

where

28 $$\begin{array}{rcl}{Q}_{1} & = & -{c}_{7}{\gamma }_{0}^{3}+({c}_{6}{c}_{10}+{c}_{7}{c}_{8}){\gamma }_{0},\\ {Q}_{2} & = & ({c}_{10}-{c}_{7}{c}_{9}){\gamma }_{0}^{2}-{c}_{5}{c}_{10},\\ {Q}_{3} & = & {\gamma }_{0}^{4}-({c}_{6}{c}_{9}+{c}_{5}+{c}_{8}){\gamma }_{0}^{2}+{c}_{5}{c}_{8},\end{array}$$

and the coefficients *B*_i_ are given by

29 $$\begin{array}{rcl}{B}_{0} & = & {c}_{5}({c}_{8}{c}_{11}-{c}_{10}{c}_{13}),\\ {B}_{1} & = & {c}_{5}({c}_{8}+{c}_{11})+{c}_{6}({c}_{9}{c}_{11}+{c}_{10}{c}_{12})+{c}_{7}({c}_{8}{c}_{12}+{c}_{9}{c}_{13})+{c}_{8}{c}_{11}-{c}_{10}{c}_{13},\\ {B}_{2} & = & {c}_{6}{c}_{9}+{c}_{7}{c}_{12}+{c}_{5}+{c}_{8}+{c}_{11}.\end{array}$$

The complete solutions of the system appeared in Eq. (27) of the considered physical quantities bound as *x* → ∞ will be on the form

30 $$\{{u}^{\ast },\,{v}^{\ast },{\theta }^{\ast }\}(x)=\mathop{\sum }\limits_{j=1}^{3}\{{H}_{j},{H}_{j}{\rm{{\prime} }},{H}_{j}{\rm{{\prime} }}{\rm{{\prime} }}\}{{\rm{e}}}^{-{\beta }_{j}x}+\{{\bar{Q}}_{1},{\bar{Q}}_{2},{\bar{Q}}_{3}\}g(y,t)\,\exp (\,-{\gamma }_{0}x),$$

where *H*_*j*_, ${H}_{j}{\rm{{\prime} }}$ and ${H}_{j}{\rm{{\prime} }}{\rm{{\prime} }}$ are the integration parameters and *β*_*j*_ (*j* = 1, 2, 3) are the +ve roots of the characteristic equations

31 $${\beta }_{j}^{6}-{B}_{2}{\beta }_{j}^{4}+{B}_{1}{\beta }_{j}^{2}-{B}_{0}=0.$$

The roots *β*_*j*_ are given respectively by

32 $$\begin{array}{rcl}{\beta }_{1} & = & \frac{1}{\sqrt{6{B}_{3}}}\sqrt{2{B}_{2}{B}_{3}+4({B}_{2}^{2}-3{B}_{1})+{B}_{3}^{2}},\\ {\beta }_{2,3} & = & \frac{1}{2\sqrt{3{B}_{3}}}\sqrt{4{B}_{2}{B}_{3}-4(1\pm {\rm{i}}\sqrt{3})({B}_{2}^{2}-3{B}_{1})-(1\mp {\rm{i}}\sqrt{3}){B}_{3}^{2}},\end{array}$$

in which

33 $$\begin{array}{rcl}{B}_{3}^{3} & = & 108{B}_{0}+8{B}_{2}^{3}-36{B}_{1}{B}_{2}+12{B}_{4},\\ {B}_{4}^{2} & = & 81{B}_{0}^{2}+12{B}_{1}^{3}-54{B}_{0}{B}_{1}{B}_{2}+12{B}_{0}{B}_{2}^{3}-3{B}_{1}^{2}{B}_{2}^{2}.\end{array}$$

Also, the parameter ${\bar{Q}}_{i}$ in Eq. (30) can be represented as

34 $${\bar{Q}}_{i}=\frac{{Q}_{i}}{{\gamma }_{0}^{6}-{B}_{2}{\gamma }_{0}^{4}+{B}_{1}{\gamma }_{0}^{2}-{B}_{0}},\,i=1,2,3.$$

The relations between the parameters ${H}_{j}$, ${H}_{j}{\rm{{\prime} }}$ and ${H}_{j}{\rm{{\prime} }}{\rm{{\prime} }}$ can be obtained by using Eq. (30) into the equations of motion appeared in Eqs. (22) and (23):

35 $$\{{H}_{j}^{{\rm{{\prime} }}},{H}_{j}^{{\rm{{\prime} }}}\}=\{{\alpha }_{j}^{1},{\alpha }_{j}^{2}\}{H}_{j},\,j=1,2,3,$$

where

36 $${\alpha }_{j}^{1}=\frac{({c}_{10}-{c}_{7}{c}_{9}){\beta }_{j}^{2}+{c}_{5}{c}_{10}}{{\beta }_{j}[{c}_{6}{c}_{10}-{c}_{7}({c}_{8}+{\beta }_{j}^{2})]},\,\,{\alpha }_{j}^{2}=\frac{{\beta }_{j}^{4}+({c}_{5}+{c}_{8}-{c}_{6}{c}_{9}){\beta }_{j}^{2}+{c}_{5}{c}_{8}}{{\beta }_{j}[{c}_{6}{c}_{10}-{c}_{7}({c}_{8}+{\beta }_{j}^{2})]},\,\,j=1,2,3.$$

Therefore, the displacements and temperature are given in their final form as

37 $$\begin{array}{c}\{u,v,\theta \}=\mathop{\sum }\limits_{j=1}^{3}{H}_{j}\{1,{\alpha }_{j}^{1},{\alpha }_{j}^{2}\}\exp (\omega t-{\beta }_{j}x+{\rm{i}}by)\\ \,\,\,\,+\{{\bar{Q}}_{1},{\bar{Q}}_{2},{\bar{Q}}_{3}\}\bar{g}(y,t)\,\exp (-{\gamma }_{0}x),\end{array}$$

where

38 $$\bar{g}(y,t)=\frac{{I}_{0}{\gamma }_{0}{q}_{t}}{2\pi {r}^{2}{t}_{0}^{3}{\varpi }_{1}}\exp \left(-\frac{{y}^{2}}{{r}^{2}}-\frac{t}{{t}_{0}}\right).$$

In addition, the stresses in their final form may be simplified as

39 $$\begin{array}{rcl}\{{\sigma }_{11},{\sigma }_{12},{\sigma }_{22}\} & = & \mathop{\sum }\limits_{j=1}^{3}{H}_{j}\{{A}_{j}^{1},{A}_{j}^{2},{A}_{j}^{3}\}\exp (\omega t-{\beta }_{j}x+{\rm{i}}by)\\  &  & -\{{\hat{Q}}_{1},{\hat{Q}}_{2},{\hat{Q}}_{3}\}\bar{g}(y,t)\exp (-{\gamma }_{0}x),\end{array}$$

where

40 $$\begin{array}{cccccc}{A}_{j}^{1} & = & -{\beta }_{j}+{\rm{i}}b{c}_{1}{\alpha }_{j}^{1}-{\alpha }_{j}^{2}, & \,{\hat{Q}}_{1} & = & {\gamma }_{0}{\bar{Q}}_{1}+{\bar{Q}}_{3}+\frac{2{c}_{1}y}{{r}^{2}}{\bar{Q}}_{2},\\ {A}_{j}^{2} & = & {c}_{1}({\rm{i}}b-{\beta }_{j}{\alpha }_{j}^{1}), & \,{\hat{Q}}_{2} & = & {c}_{1}(\frac{2y}{{r}^{2}}{\bar{Q}}_{1}+{\gamma }_{0}{\bar{Q}}_{2}),\\ {A}_{j}^{3} & = & -{c}_{1}{\beta }_{j}+{\rm{i}}b{\alpha }_{j}^{1}-{\alpha }_{j}^{2}, & \,{\hat{Q}}_{3} & = & {c}_{1}{\gamma }_{0}{\bar{Q}}_{1}+{\bar{Q}}_{3}+\frac{2y}{{r}^{2}}{\bar{Q}}_{2}.\end{array}$$

## Thermomechanical conditions

The boundary conditions on the surface of the half-space medium can be applied to get the parameters *H*_*j*_ (*j* = 1, 2, 3). The positive exponentials are taken boundless at infinity in this physical problem. Concerning the mechanical boundary conditions, we have:

(i) The traction load can be applied on the plane surface *x* = 0 and takes the value *σ*_0_ in normal direction:

41 $${\sigma }_{11}(0,y,t)=f(y,t)={\sigma }_{0}\,\exp (\omega t+{\rm{i}}by).$$

(ii) The tangent traction is free

42 $${\sigma }_{12}(0,y,t)=0.$$

(iii) The thermal boundary on the surface *x* = 0 is uniform. That is

43 $$\theta (0,y,t)={\theta }_{0}.$$

Therefore, using Eqs. (37)_3_, (39)_1_ and (39)_2_ for *θ*, *σ*_11_ and *σ*_12_, respectively, the parameters *H*_*j*_ can be determinate by solving the following relations:

44 $$\{\begin{array}{c}{H}_{1}\\ {H}_{2}\\ {H}_{3}\end{array}\}={[\begin{array}{ccc}{A}_{1}^{1} & {A}_{2}^{1} & {A}_{3}^{1}\\ {A}_{1}^{2} & {A}_{2}^{2} & {A}_{3}^{2}\\ {\alpha }_{1}^{2} & {\alpha }_{2}^{2} & {\alpha }_{3}^{2}\end{array}]}^{-1}\{\begin{array}{c}{\sigma }_{0}-{\hat{Q}}_{1}g(y,t)\\ -{\hat{Q}}_{2}g(y,t)\\ {\theta }_{0}-{\bar{Q}}_{3}g(y,t)\end{array}\}.$$

## Validation and applications

Some applicable examples will be presented to put into suggestion the impact of different models on the variable quantities. The material properties of the annular disk are mentioned according to the following values of parameters:

$$\lambda =7.76\times {10}^{10}\,{{\rm{Nm}}}^{-2}$$,$$\mu =3.86\times {10}^{10}\,{{\rm{Nm}}}^{-2}$$,$$k=386\,{{\rm{Wm}}}^{-1}\,{{\rm{K}}}^{-1}$$,$$\rho =8954\,{\rm{kg}}\,{{\rm{m}}}^{-3}$$,$${\alpha }_{t}=1.78\times $$$${10}^{-5}\,{{\rm{K}}}^{-1}$$, $${C}_{e}=383.1\,{\rm{J}}\,{{\rm{kg}}}^{-1}\,{{\rm{K}}}^{-1}$$, *T*_0_ = 293 K, k^*^ = 1.5, $${I}_{0}={10}^{2}\,{\rm{J}}\,{{\rm{m}}}^{-2}$$, $$r=0.2\,{\rm{\mu }}m$$, $${\gamma }_{0}=25\,{{\rm{m}}}^{-1}$$, $${t}_{0}=10\,{\rm{ns}}$$, $${\theta }_{0}=10$$, *σ*_0_ = 1.

**Figure 2:**
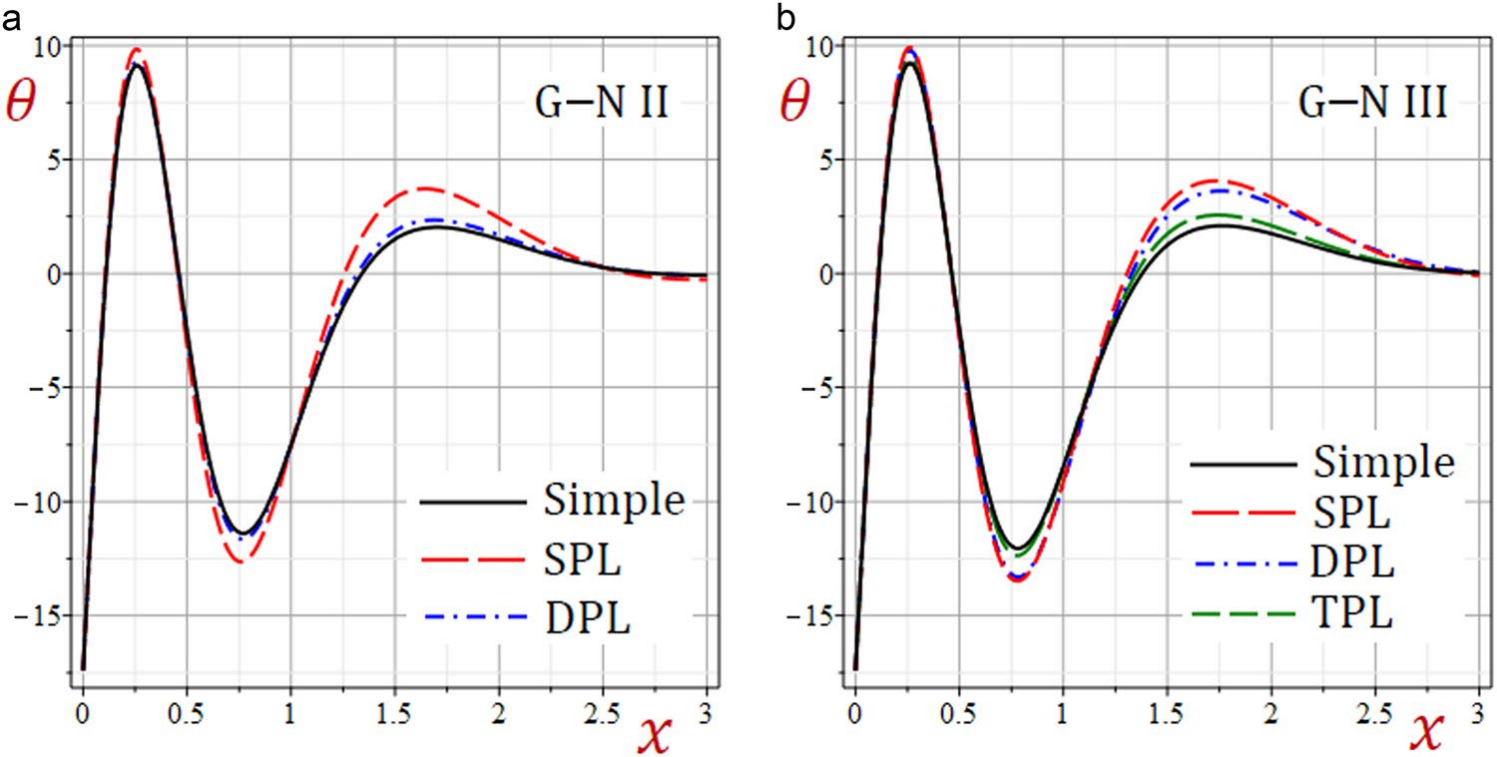
Effect of G–N models on the temperature *θ* of the medium.

**Figure 3:**
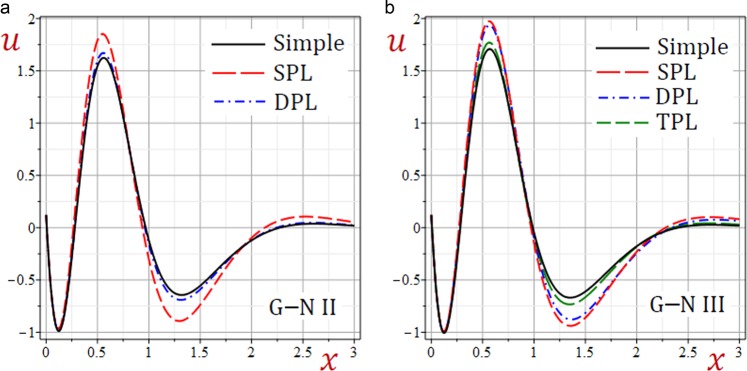
Effect of G–N models on the displacement *u* of the medium.

**Figure 4:**
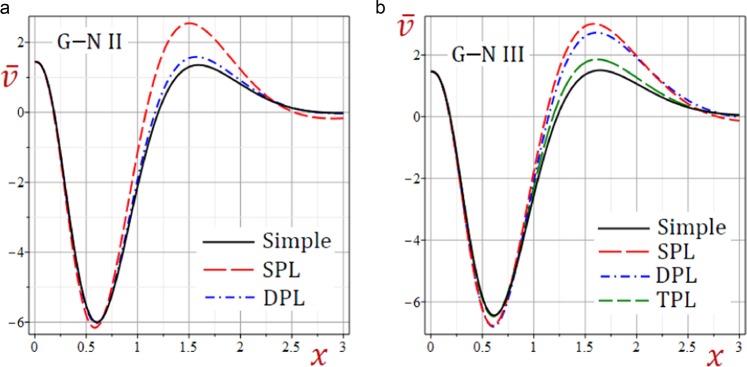
Effect of G–N models on the displacement *v* of the medium.

**Figure 5:**
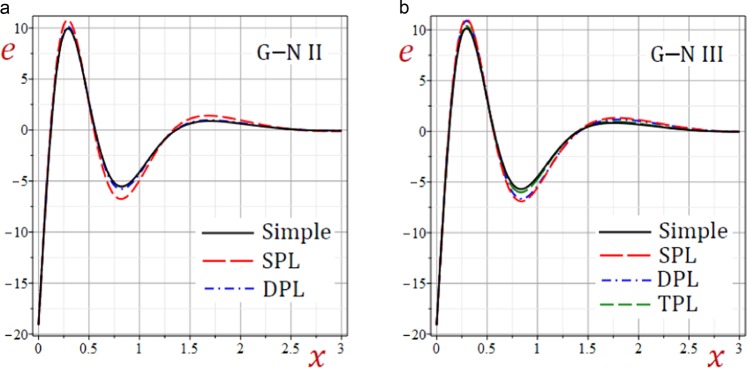
Effect of G–N models on the dilatation *e* of the medium.

**Figure 6:**
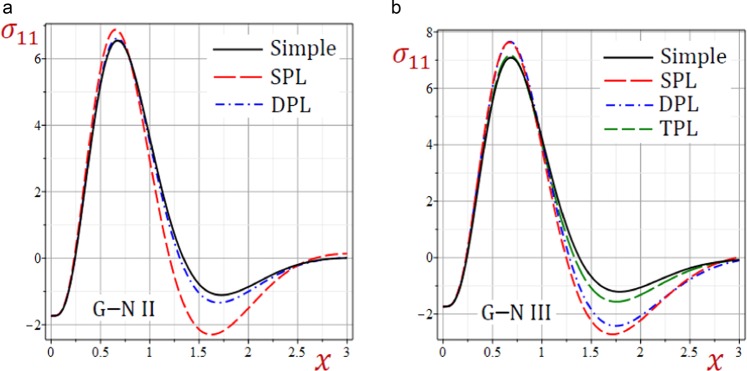
Effect of G–N models on the stress *σ*_11_ in the medium.

**Figure 7:**
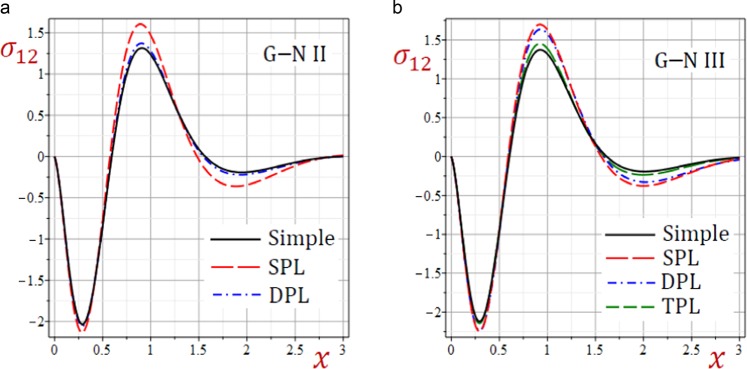
Effect of G–N models on the stress *σ*_12_ in the medium.

**Figure 8:**
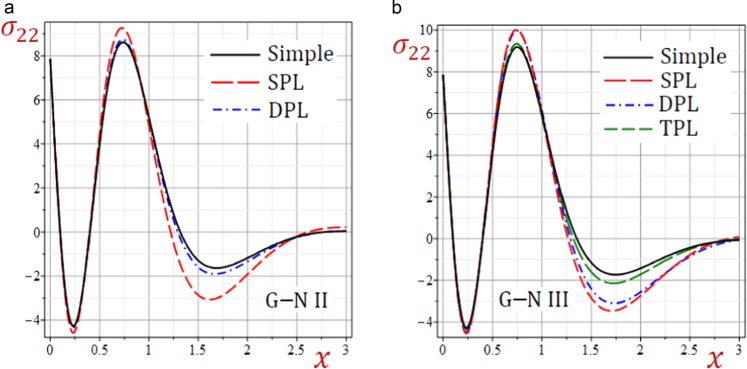
Effect of G–N models on the stress *σ*_22_ in the medium.

**Figure 9:**
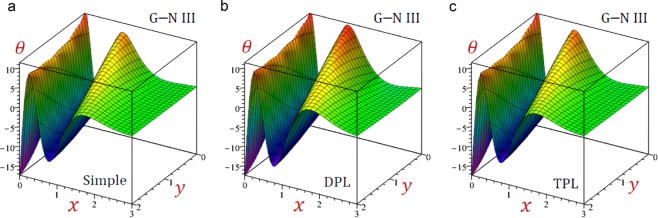
3D distributions of the temperature of the medium using G–N III theory.

**Figure 10:**
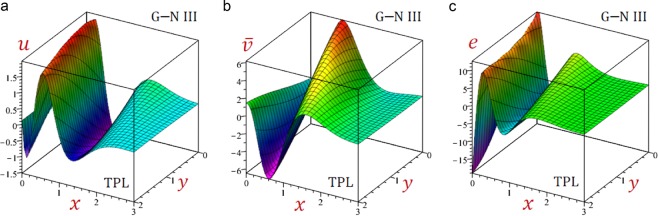
3D distributions of displacements and dilatation of the medium using TPL G–N III theory.

For convenience, the real values of the field quantities have been adopted to represent the outcomes. Numerical results are obtained for $\omega =1.9+2.9{\rm{i}}$, ${\tau }_{q}=0.2$, ${\tau }_{\theta }=0.15$, ${\tau }_{\vartheta }=0.1$, $b=\frac{\pi }{3}$ and *t* = 0.3. Results of all variable field quantities of the half-space due to G–N II, SPL G–N II and DPL G–N II thermoelasticity theories are reported in Table 1 for a fixed *y* = 2 and different values of *x*. That is *θ*(1.5), *u*(0.6), $\bar{v}=10\,v(1.5)$, *e*(0.5), *σ*_11_(0.7), *σ*_22_(0.7) and *σ*_12_(1.0). Similar results of the variable field quantities based on G–N III, SPL G–N III, DPL G–N III, and TPL G–N III thermoelasticity theories are reported in Table 2. Additional sample 1D and 2D graphs in the half-space are plotted in Figs. 1–10 to investigate the effect of all models on the field quantities. The results reported in both tables will serve as benchmarks for other investigators. It is concluded from these tables that:

- The simple G–N II yields the smallest temperature *θ*, displacements *u* and $\bar{v}$, stresses, *σ*_22_ and *σ*_12_ while it yields the greatest dilatation *e*.
- The modified G–N II and G–N III may be applied with a number of terms reaches 6. In fact, *N* = 5 may be enough to get accurate results.
- All modified, SPL G–N II, DPL G–N II, SPL G–N III, DPL G–N III, and TPL G–N III models yield smaller variable quantities comparing to the simple G–N II and G–N III models.
- For all modified SPL, DPL or TPL models the results are slightly decreasing with the increase in a number of terms. The decreasing amounts may be insensitive when *N* ≥ 5.
- For the modified G–N II and III, the DPL model gives quantities greater than those of the SPL model.
- For the modified G–N III, the TPL model gives quantities greater than those of the DPL model.

**Table 1:** Effect of G–N II thermoelasticity theories on all quantities of the medium.

		*θ*	*u*	$\bar{{\boldsymbol{v}}}$	*e*	*σ*_11_	*σ*_12_	*σ*_22_
	Simple	1.512372	1.591605	1.301214	2.757593	6.519950	1.235728	8.506570
SPL	*N* = 1	2.952816	1.706902	2.180816	2.363967	6.482731	1.371582	8.856741
	*N* = 2	3.365649	1.780184	2.523733	2.462019	6.687988	1.456905	9.142401
	*N* = 3	3.354514	1.794955	2.563222	2.534689	6.766222	1.473206	9.206851
	*N* = 4	3.332676	1.794690	2.554985	2.544888	6.773250	1.472594	9.207583
	*N* = 5	3.329662	1.794288	2.552766	2.544662	6.772398	1.472103	9.206117
	*N* = 6	3.329632	1.794242	2.552618	2.544470	6.772178	1.472051	9.205920
DPL	*N* = 1	1.772513	1.608112	1.461717	2.662211	6.486078	1.255494	8.543088
	*N* = 2	1.877064	1.627437	1.550205	2.668080	6.534818	1.275813	8.619095
	*N* = 3	1.871791	1.633587	1.560353	2.692088	6.566787	1.281716	8.648939
	*N* = 4	1.862517	1.633615	1.556018	2.697346	6.570842	1.281590	8.650457
	*N* = 5	1.861188	1.633413	1.554971	2.697398	6.570431	1.281372	8.649705
	*N* = 6	1.861193	1.633386	1.554917	2.697302	6.570297	1.281346	8.649577

**Table 2:** Effect of G–N III thermoelasticity theories on all quantities of the medium.

		*θ*	*u*	$\bar{{\boldsymbol{v}}}$	*e*	*σ*_11_	*σ*_12_	*σ*_22_
	Simple	1.250421	1.688300	1.367425	3.175144	7.078673	1.319450	9.003136
SPL	*N* = 1	2.825331	1.852343	2.541629	3.028455	7.242929	1.524866	9.502878
	*N* = 2	3.155114	1.933443	2.942543	3.210221	7.510762	1.621049	9.803943
	*N* = 3	3.084529	1.945332	2.958523	3.285629	7.590957	1.631951	9.859016
	*N* = 4	3.054645	1.944116	2.942560	3.292630	7.595473	1.629742	9.857132
	*N* = 5	3.051885	1.943642	2.939768	3.291854	7.594193	1.629145	9.855498
	*N* = 6	3.052016	1.943601	2.939672	3.291644	7.593959	1.629105	9.855321
DPL	*N* = 1	2.417869	1.836434	2.323061	3.145307	7.315904	1.497349	9.482311
	*N* = 2	2.627620	1.903557	2.628105	3.306441	7.553721	1.573455	9.737820
	*N* = 3	2.553403	1.913119	2.629475	3.370931	7.623653	1.581172	9.785402
	*N* = 4	2.526651	1.911881	2.613832	3.376771	7.627193	1.579016	9.783421
	*N* = 5	2.524440	1.911445	2.611392	3.376033	7.625950	1.578488	9.781894
	*N* = 6	2.524596	1.911409	2.611337	3.375843	7.625733	1.578456	9.781732
TPL	*N* = 1	1.640052	1.713541	1.627332	3.071965	7.038972	1.353852	9.052592
	*N* = 2	1.774255	1.739417	1.757544	3.093316	7.106089	1.382411	9.147667
	*N* = 3	1.756790	1.746352	1.766877	3.123781	7.144858	1.388677	9.180943
	*N* = 4	1.743602	1.746103	1.759818	3.128889	7.148787	1.388068	9.181799
	*N* = 5	1.742150	1.745849	1.758455	3.128719	7.148177	1.387782	9.180894
	*N* = 6	1.742212	1.745821	1.758414	3.128602	7.148027	1.387757	9.180764

**Figure 11:**
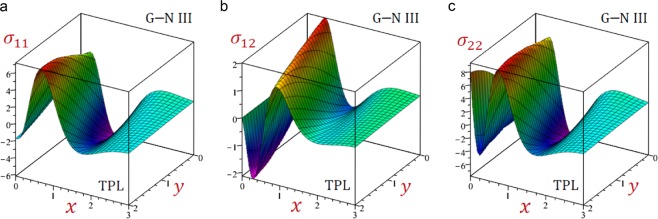
3D distributions of stresses in the medium using TPL G–N III theory.

Now, Figs. 2–11 are presented as a sample to illustrate the effect of all models on the temperature, displacements, dilatation, and stresses along the *x*-axis of the medium. Figure 2 presents the temperature distribution as waves that begin with negative values and ends with zero as *x* increases. The single-phase-lag (SPL) Green–Naghdi II and III models give the temperature *θ* with the largest amplitude. However, the simple G–N II and III models give the temperature *θ* with the smallest amplitude. For G–N III, the dual-phase-lag (DPL) model yields temperature *θ* with amplitude intermediates those of the SPL and triple-phase-lag (TPL) models. Also, the TPL model yields temperature *θ* with amplitude intermediates those of the DPL and the simple ones. The relative errors between models increase at the peak points of the temperature wave.

Figures 3–5 present the distributions of the displacements *u*, $\bar{v}$ and the dilatation *e* along the *x*-axis of the medium. The displacement-waves begin with positive values and ends with zero as *x* increases while dilatation-wave begins with negative values and ends with zero as *x* increases. The SPL G–N II and III models give the displacements *u*, $\bar{v}$ and dilatation *e* with the largest amplitudes. However, the simple G–N II and III models give the displacements *u*, $\bar{v}$ and dilatation *e* with the smallest amplitudes. For G–N III, the DPL model yields displacements *u*, $\bar{v}$ and dilatation *e* with amplitudes intermediate those of the SPL and TPL models. Also, the TPL model yields displacements *u*, $\bar{v}$ and dilatation *e* with amplitudes intermediate those of the DPL and the simple ones. The relative errors between models increase at the peak points of the displacement and dilatation waves.

Figures 6–8 present the distributions of all stresses along the *x*-axis of the medium. The in-plane normal stress-waves *σ*_11_ begin with negative values while the in-plane longitudinal stress-waves *σ*_22_ begin positive values and the in-plane tangential stress-waves *σ*_12_ begin with zero values. All stresses vanish as *x* increases. The SPL G–N II and III models give stresses with the largest amplitudes. However, the simple G–N II and III models give stresses with the smallest amplitudes. For G–N III, the DPL model yields stresses with amplitudes intermediate those of the SPL and TPL models. Also, the TPL model yields stresses with amplitudes intermediate those of the DPL and the simple ones. The relative errors between models increase at the peak points of the stress waves.

Finally, Figs. 9–11 present the 3D distributions of all field quantities of the medium using G–N III theory. The maximum temperature due to the simple, DPL and TPL models occurs at the origin point (0, 0) while the minimum one occurs at the point (0, 2). The maximum displacements *u*, $\bar{v}$ and dilatation *e* occur at different positions when *y* = 0. The maximum normal *σ*_11_ and longitudinal *σ*_22_ stresses occur at different positions when y = 2 while the maximum tangential stress *σ*_12_ occurs when *y* = 0. The wave amplitude for all quantities is decreasing as *x* increases. These figures are very important to study the dependence of the physical quantities on the 2D components of the distance.

## Conclusions

This article presents analytical solutions for generalized thermoelastic interaction with multi thermal relaxations on a half-space subjected thermal loading due to laser pulse. The nonhomogeneous basic equations of the mathematical model are derived. The surface of the half-space is taken to be traction free in the tangential direction with uniform heat and traction in the normal direction. The system of two differential coupled equations is solved using the normal mode approach, and the temperature, displacements, dilatation, and stresses are obtained for the thermoelastic interaction of the medium. The modified Green and Naghdi theories of types II and III are presented to get novel and accurate models of single-, dual-, and three-phase-lag of multi terms. The third phase-lag is included in the Green and Naghdi theory. This process may help experimental scientists working in the area of computational wave propagation. Some results are tabulated to serve as benchmark results for future comparisons with other investigators. The reported and illustrated results show that the simple G–N II and III models yield the largest values of all field quantities. The single-phase-lag model gives the smallest values. However, the dual-phase-lag model yield results that intermediate those of the simple and single-phase-lag Green-Naghdi II models. Finally, the dual-phase-lag and the tree-phase-lag models yield results that intermediate those of the simple and single-phase-lag Green-Naghdi III models. In fact, one can easily see that the different models have great effects on all field quantities which supports the physical fact.
